# The Association Between Work Passion and Research Ability in Male Nurses: A Multicenter Cross-Sectional Study in China

**DOI:** 10.1155/jonm/8436449

**Published:** 2025-09-18

**Authors:** Tengfei Liu, Shu Yan, Yang Yu, Rui Yang, Xiangyu Zhang, Xingyao Wu, Xinzhu Li, Yihan Lv, Chunyang Wang, Ziyi Ding, Ye Zhao, Haishui Shi

**Affiliations:** ^1^Hebei Key Laboratory of Early Life Health Promotion (SZX202419), Hebei Medical University, Shijiazhuang 050031, China; ^2^Infection Management Service, The First Hospital Affiliated of Hebei North University, Zhangjiakou 075000, China; ^3^Neuroscience Research Center, Institute of Medical and Health Science, Hebei Medical University, Shijiazhuang 050017, China; ^4^The Key Laboratory of Neural and Vascular Biology, Hebei Medical University, Ministry of Education, Shijiazhuang 050017, China; ^5^Nursing School, Hebei Medical University, Shijiazhuang 050031, China; ^6^The Fourth Hospital of Hebei Medical University, Shijiazhuang 050000, China

**Keywords:** academic achievement, male nurses, research ability, work passion

## Abstract

**Aim:** To investigate the relationship between work passion and research ability in male nurses from China.

**Background:** Male nurses are vital to global nursing, yet face severe shortages, occupational stress, and research constraints. While work passion significantly impacts job performance across fields, its role in shaping male nurses' research ability remains underexplored. Filling this gap is critical for addressing systemic barriers and fostering their academic contributions, ultimately advancing nursing science. The advancement of the nursing discipline heavily relies on frontline nursing researchers. However, the global shortage of nursing staff is particularly severe among male nurses and their participation in research is limited. Existing studies focus on female nurses, neglecting systemic barriers like funding, mentorship, and stereotypes that hinder male nurses' academic growth. This study addresses this gap by examining how work passion shapes male nurses' research ability, offering insights to foster their professional development and scientific contributions.

**Methods:** This multicenter cross-sectional study, supported by the Provincial Nursing Association by using a convenience sampling method, surveyed 438 male nursing staff across 87 provincial hospitals from Hebei Province, including 39 tertiary levels A hospitals and 7 medical college-affiliated hospitals. Data collection involved demographic information, the Work Passion Scale (WPS), and the Nursing Research Capability Scale (NRCS). Data were analyzed using Pearson correlation, *T*-tests, and one-way ANOVA.

**Results:** Results show that the primary barrier to scientific research identified was difficulty in selecting research topics. Positive correlations were found between both dimensions of work passion (harmonious and obsessive) and all dimensions of nursing research ability. Male nurses with higher work passion scores demonstrated significantly higher research ability compared with those with lower work passion.

**Conclusion:** This study highlights the significant challenges faced by male nurses in the 87 provincial hospitals surveyed in a scientific research, such as difficulties in topic selection, writing skills, financial support, and mentorship. Our findings reveal that reduced work passion negatively impacts their research development. Hospital managers should implement targeted policies to enhance work passion, such as providing research training, financial support, and mentorship programs. By addressing these issues, we can foster a more supportive environment that promotes both the scientific research ability and the overall professional development of male nurses.

## 1. Introduction

Nursing research includes systematic investigation and evaluation of clinical nursing practice, nursing management, and nursing education [1]. At the same time, nursing research is an integral part of professional nursing, and study findings can be used to develop and change nursing practice [2]. With the rapid development of higher nursing education and the increasing demand for clinical nursing, the development of the nursing discipline must be supported by high-quality clinical nursing and scientific research [3, 4]. Currently, the research capacity in the field of nursing worldwide is still insufficient [2, 5], but research capabilities of clinical nurses have not significantly improved over the years, so it is crucial to cultivate the research ability of nursing staff [6, 7]. In the past two decades, nurses in Chinese academic institutions and large hospitals have been committed to improving their research ability, such as improving nurses' research awareness, involving nurses in research, and improving research productivity [8]. Frontline nursing staff are the backbone of nursing research, and their research ability directly affects the development of the nursing discipline. Cultivating nurses' research ability is conducive to promoting the comprehensive development of the nursing discipline and meeting the increasing and multilevel nursing needs in clinical practice [9, 10].

However, cultivating nursing research ability has been recognized as a major challenge worldwide [11–13], especially in China, which has vast medical resources and a large nursing workforce [14]. A study in China revealed that nurses' research capabilities are influenced by various factors, including age, education level, technical titles, and administrative positions [15]. Issues such as resource constraints [16], organizational barriers [17], and the complexity of research topics and methods [18, 19] hinder the development of nursing research in China. The accumulation of these negative factors can reduce nurses' passion for work and research [20, 21]. In all fields, work passion is crucial for the quality of employees' work [22]. Meanwhile, work passion can affect the overall performance of nurses in their work [23]. Work passion in the field of nursing includes positive emotional experiences, patient care, professional development, and teamwork, which enhance overall happiness [24, 25]. Evidence shows that work passion is positively correlated with creativity [26], and intrinsic work passion significantly improves employee creativity [27]. It means that good work passion may positively impact the research ability of nursing staff. Vallerand and Houlfort's dual passion model divides work passion into two dimensions: harmonious and obsessive [22]. High work passion significantly improves the quality of nursing services and patient satisfaction [24, 28]. On the other hand, increased workload and research pressure can dampen nurses' work enthusiasm. Therefore, a deep understanding of the impact of work passion on nursing research ability is of guiding significance for strategies to cultivate the research ability of nursing staff.

With the development of nursing and the diversification of societal demands for nursing, an increasing number of men are entering the nursing profession, becoming an important part of the diversified development of the nursing industry. After the establishment of the Male Nurses Working Committee by the Chinese Nursing Association in 2014, male nurses working organizations have been established in various provinces. The research capabilities of male nurses are particularly noteworthy [29]. According to data from China's National Health Commission, as of the end of 2023, the total number of registered nurses nationwide reached 5.63 million, with over 80% having a college diploma or above [30]. In addition, male nurses accounted for about 3.4%, far lower than the 10% in developed countries [31]. Compared with female nurses, male nurses' physical and physiological advantages in high-intensity work and research may lead to higher work efficiency and better physical and mental health [29]. A descriptive qualitative study found that patients perceived male nurses as respectful, considerate, good listeners, fair, and supportive [32]. However, the recognition of male nurses by society is still very low, and male nurses may encounter different stereotypes in their work [33, 34]. Despite the surging demand from the society, male nurses still share the problem of low career identification, higher mental stress, and lack of job planning due to the impact of traditional concept, improper self-awareness, and low income [35–38]. It has been reported that male nurses students have a high rate of switching majors during their studies, and after graduation, they often choose non-nursing positions, leading to a high rate of occupational turnover [39]. The unique challenges and complexity of the nursing environment make male nurses more vulnerable to occupational stress, which directly threatens their mental health and may further affect their work enthusiasm and research willingness [40, 41].

Therefore, this study mainly aimed to investigate the relationship among male nurses from Hebei Province in China between work passion and research ability, as well as the potential influence of demographic information on male nurses. Effective management measures are proposed to reduce occupational stress among male nurses, enhancing their work engagement and research willingness. This study aims to provide valuable insights for improving the work passion and research ability of the male nursing workforce, ultimately contributing to the nursing discipline's overall development and improving the healthcare system's overall effectiveness.

## 2. Materials and Methods

### 2.1. Participants

This study utilized online and social media channels to assess the work passion and research abilities of male nurses. Supported by the School of Nursing at Hebei Medical University and the Hebei Nursing Association, questionnaires were distributed to male nursing staff in 87 hospitals across Hebei Province from November to December in 2023, including 39 tertiary level A hospitals and 7 medical college-affiliated hospitals. The inclusion criteria were as follows: (1) nurses employed in hospitals during the survey period; (2) nurses with valid nursing licenses; and (3) nurses who consented to participate in the study. Prior to data collection, all participants were informed that their responses would remain anonymous and confidential.

For an one-way ANOVA test design with an anticipated medium effect size of 0.25, a power level of 0.95, and *p* < 0.05 for five groups across ten measurement times, a priori sample size calculation using G∗Power 3.1.9.6 software suggested a minimum sample size of 305 participants. For a correlation analysis design with an anticipated medium effect size of 0.25, a power level of 0.95, and *p* < 0.05 for two groups across ten measurement times, the minimum required sample size was calculated to be 197 participants. The analysis focused on male nurses aged 18–65, excluding responses that were excessively short or lengthy. All participants provided informed consent in accordance with medical ethical standards before participating in the online survey.

### 2.2. Assessment

The online survey tool “Questionnaire Star” was used to design and administer the questionnaire. The qualitative data collection process, conducted through this platform, spanned approximately 130–600 s. The lower limit was based on the fastest completion time by 15 male caregiver volunteers. To address potential impacts on questionnaire quality with prolonged duration, medical education experts from a Medical University assessed and established the upper time limit, which was set at 600 s. In the process of online scale evaluation, we set interference questions (such as answering an additional mirror question of a question) to remind subjects to read the questions carefully and answer them appropriately. When the difference between the absolute value of the subject's mirror question and the original question is less than or equal to 2, we will exclude the questionnaire, which will help us significantly improve the quality and effectiveness of questionnaire recovery.

Participants could save and review responses before submission. Collected demographic information including age, education, years of operation, job title, position, unit type, academic part-time job status, research project hosting status, publication status, journal reviewer status, academic exchange participation, technological awards, research willingness, and main factors restricting scientific research. Subjects were evaluated using the Work Passion Scale (WPS) [22, 42] and the Nurse Research Capacity Scale (NRCS) [43].

Data analysis included Pearson correlation analysis, confidence testing, and one-way ANOVA. All scales were referenced from authoritative journals and selected for their high reliability and evaluation quality. Additionally, our team engaged three bilingual doctors to conduct translations and reverse-translations of relevant scales [44–46], following the procedure outlined by Brislin. In this process, we asked three bilingual doctors to fully understand the original meaning of each question on the original English scales and then translate them into Chinese sentence by sentence on this basis. Finally, we reverse-translate the translated Chinese scale into English again and compare whether there are differences in expression between the original English and the reverse-translated English scales. Two pedagogy experts were consulted to review and refine the scales' content based on the given context [26].

#### 2.2.1. Demographic Information

Concerning demographic information, the questionnaire assessed participants' age (1 = under 30 years old, 2 = 31–35, 3 = 36–40, 4 = 41–50, and 5 = over 50), educational background (1 = Below Associate degree, 2 = Associate degree, 3 = Bachelor degree, 4 = Master degree, and 5 = Doctor degree), entire period of actual operation (1 = Less than 3 years, 2 = 3–5 years, 3 = 5–10 years, 4 = 10–15 years, and 5 = More than 15 years), job title (1 = None, 2 = Junior title, 3 = Medium-grade title, 4 = Associate senior title, and 5 = Senior title), academic part-time job status (1 = None, 2 = Other committee member, 3 = Municipal committee member and above, 4 = Provincial committee members and above, and 5 = National committee members and above), research project hosting status (1 = None, 2 = Internal unit level, 3 = Municipal level, 4 = Provincial level, and 5 = National level), published papers status (1 = None, 2 = Other journal, 3 = Science and technology core journal of China, 4 = Chinese core journal of China, and 5 = SCI journal), academic journal reviewer status (1 = None, 2 = Other journal, 3 = Science and technology core journal of China, 4 = Chinese core journal of China, and 5 = SCI journal), academic exchange participation status (1 = None, 2 = Other, 3 = Provincial, 4 = National, and 5 = International), technological awards status (1 = None, 2 = Internal unit, 3 = Municipal, 4 = Provincial, and 5 = National), and the main factors that restrict scientific research activities (A = Age, B = Entire period of actual operation, C = Educational background, D = Job title, E = Marital status, F = Scientific research topic, G = Scientific research design, H = Data collection, I = Statistical skill, J = Writing ability, K = Scientific research thinking, L = Working load, M = Financial support and the help of mentors, and N = The importance that leaders attach to research).

#### 2.2.2. Working Passion Scale (WPS)

The WPS is a self-report instrument designed to assess the working passion of male nurses subjectively. Developed by Vallerand et al. [22], the scale underwent cuts and revisions, resulting in two dimensions and fourteen items. It evaluates two factors contributing to the professional identity of doctors: harmonious passion and obsessive passion. The original and revised versions of WPS have demonstrated favorable measurement characteristics, encompassing internal consistency, reliability, as well as convergence and divergence [22]. The version we use is translated by Song Yahui et al. [42] from the scale developed by Valleran et al., which is commonly used in China. The scale operates within a range of 14–98 points, with each item rated on a scale of 1 (strongly disagree) to 7 (strongly agree). A higher score on the scale indicates a stronger working passion for nursing. Following reliability analysis, the questionnaire exhibited a good Cronbach's alpha value (*α* = 0.866).

#### 2.2.3. Nurse Research Capacity Scale (NRCS)

The NRCS is a self-report instrument designed to assess the research ability of male nurses subjectively. It was developed by Chinese scholar Ruishuang Liu [43] and revised by Yinhe Pan [47]. The scale consists of 6 dimensions and 3 items. It evaluates six factors contributing to the professional identity of doctors: problem finding ability, literature review ability, research and design capability, scientific research practice ability, data processing ability, and paper writing ability. The scale operates from 0 to 120 points, with each item rated on a scale of 0 (strongly disagree) to 4 (strongly agree). A higher score on the scale indicates a stronger nurse research capacity with nursing. Following reliability analysis, the questionnaire exhibited a good Cronbach's alpha value (*α* = 0.985).

### 2.3. Statistical Analysis

Data analysis was conducted using SPSS 21.0 software. The procedure involved initial deviation analysis using common methods. Subsequently, descriptive statistics and Pearson correlation analysis were conducted for the main variables. One-way ANOVA was used to analyze differences in research ability scores across various demographic and professional categories. *T*-tests were conducted to compare research ability scores between low and high work passion groups. Results were reported as the mean ± standard deviation for descriptive statistics, *R*-values, and *p* values for Pearson correlations and F and *p* values for ANOVA. Data on main constraints to research activities were presented in pie chart and bar chart.

## 3. Results

### 3.1. Description of the Sample

The statistics are described in terms of percentages, the mean and standard deviation of the normal distribution curve, and the median and range of the non-normal distribution curve (Min–Max). After quality assessment, we finally included 438 valid questionnaires. The mean age score was 1.95 (±1.05), the mean educational background score was 2.78 (±0.47), the mean entire period of actual operation score was 3.01 (±1.35), the mean job title score was 2.38 (±0.79), the mean academic part-time job status score was 1.5 (±1.06), the mean research project hosting status score was 1.24 (±0.69), the mean published papers status score was 1.37 (±0.87), the mean academic journal reviewer status score was 1.10 (±0.47), the mean academic exchange participation status score was 1.55 (±0.99), and the mean technological awards status score was 1.15 (±0.59).

**Figure 1:**
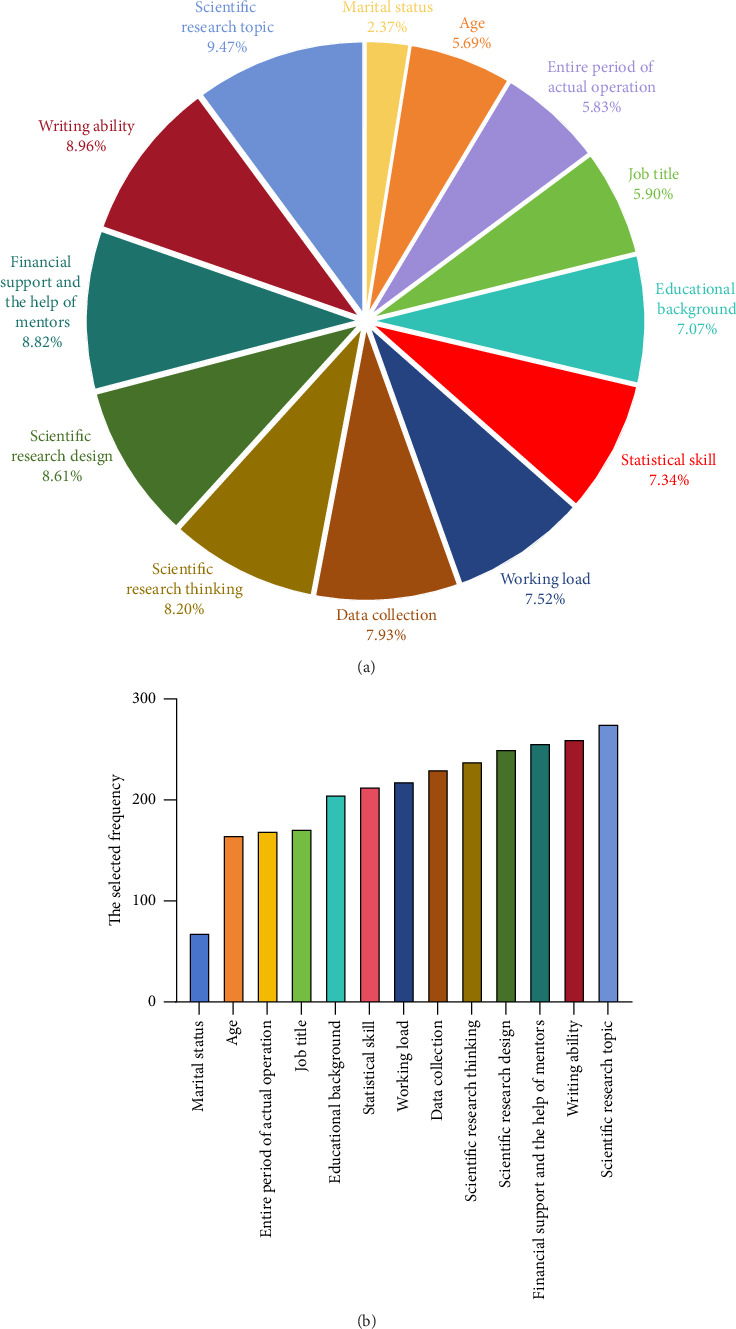
(a) The pie chart is the proportional distribution of the main factors that the subjects believe restrict the development of scientific research activities. (b) The frequency bar chart is the proportion distribution of the main factors that subjects believe restrict the development of scientific research activities.

Regarding the importance of nursing scientific research, 371 subjects (84.70%) considered it important to carry out nursing research work, and 316 subjects (72.15%) had intentions and ideas to carry out nursing research work. In the whole sample, the mean total score of WPS was 66.03 (±14.98), harmonious passion score was 38.27 (±9.59), and obsessive passion score was 27.75 (±8.80). The mean total score of NRCS was 91.85 (±28.52), problem finding ability score was 10.74 (±2.56), literature review ability score was 16.73 (±4.66), research and design capability score was 14.31 (±5.61), scientific research practice ability score was 18.15 (±6.58), data processing ability score was 14.62 (±5.55), and paper writing ability score was 17.29 (±6.95). In addition, among the main factors that restrict the development of scientific research activities, the lack of research topics (*N* = 276, 9.47%) is considered to be the most important factor, followed by the lack of writing ability (*N* = 261, 8.96%) and the lack of financial support (*N* = 257, 8.82%). Detailed assessment data of subjects are summarized in Table 1 and Figure 1.

### 3.2. Common Method Bias Test of Variables

The results of the principal component factor analysis showed that 11 factors in the total samples featured a characteristic grounding greater than 1 and that the variation explained by the first factor was only 33.04%, i.e., less than 40% of the critical standard [48]; there is no evidence of a common method bias.

### 3.3. Correlation Analysis Between WPS and NRCS

Pearson correlation analysis of WPS and NRCS scores showed that the total score of research ability and its six elements (problem finding ability, literature review ability, research and design ability, scientific research practice ability, data processing ability, and paper writing ability) were positively correlated with the total score of the work passion, harmonious passion, and compulsive passion. The correlation analysis between WPS and NRCS scores is shown in Table 2.

### 3.4. Comparison of NRCS and Its Six Dimensions Scores Between Low and High Working Passion Groups

**Figure 2:**
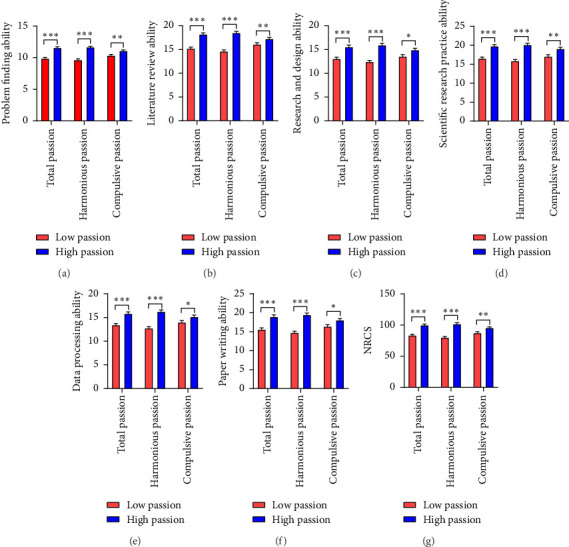
The histogram model comparing the scores of the Nurse Research Capacity Scale (NRCS) and its six dimensions in the three dimensions of total passion, harmonious passion, and forced passion between the high passion group and the low passion group. ^∗^*p* < 0.05, ^∗∗^*p* < 0.01, and ^∗∗∗^*p* < 0.001, calculated using 2-tailed bivariate correlations.

According to previous studies, we divided the subjects into a high-work-passion group and a low-work-passion group by the dichotomous method [49] and conducted an independent sample *T*-test for the NRCS and six dimensions scores of both groups. The statistics showed that the high WPS group had significantly higher scores for NCRS and its six dimensions than the low WPS group. In addition, the scores of NCRS and six dimensions of the high harmonious work passion group and high compulsive work passion group were significantly higher than those the low harmonious work passion group and low compulsive work passion group, respectively. The comparison data of the scores on the scales are summarized in Figure 2.

### 3.5. Comparison of Total NRCS Scores Between Groups of Demographic Information

In order to further explore the potential impact of demographic information on the research ability of male nurses and the differences in the total NRCS score between groups of demographic information, we divided each demographic information into five groups (e.g., score 1 equals the first group) and then conducted nonparametric tests. The analysis results show that there were significant differences in NRCS total scores of educational background, entire period of actual operation, and hospital level between the different groups. There were statistically significant differences in research project hosting status and academic journal reviewer status compared with other dimension of NRCS scores (Table 3). There was no significant difference in the total NRCS score between other demographic information groups. The comparison data of the NRCS total scores on the demographic information between groups are summarized in Table 4.

## 4. Discussion

This study, supported by the School of Nursing at Hebei Medical University and the Hebeisheng Nursing Association, utilized an online survey method to investigate the relationship between work passion and research ability among male nurses in 87 hospitals from Hebei Province. The study also examined the factors impeding scientific research and the impact of demographic information on research ability, obtaining 438 valid samples. This is the first multicenter cross-sectional study to explore the relationship between research ability and work passion among male nurses. Our study will help address the challenges male nurses face in work and research in the context of a global shortage of male nurses. This study yielded three main findings. First, total work passion and its dimensions (harmonious passion and obsessive passion) were significantly positively correlated with nursing research ability and its six dimensions. Second, the research ability scores of the high work passion group were significantly higher than those of the low work passion group. Finally, the main barriers to nursing research included difficulties in selecting research topics, lack of financial support, and challenges in research design. Overall, the findings emphasize the significant impact of work passion on the research ability of male nurses. These insights can assist hospital managers in formulating supportive policies to enhance male nurses' work passion and research ability, further promoting the comprehensive development of the nursing discipline.

Our survey found that male nurses are less active in scientific research, with many having never published articles or hosted research projects. Most have a bachelor's degree background, and the vast majority has no academic part-time jobs or academic exchanges. This indicates that male nurses have great potential to advance nursing research. Male nurses work on the front lines of clinical care, providing convenient care for male patients and bringing unique insights due to their physical strength. However, these insights have not been scientifically summarized and explored due to a lack of research ability. Therefore, enhancing the research capability of male nurses is crucial for improving the research system of the healthcare industry and advancing public health. This study is the first to use WPS and NRCS to assess the relationship between work passion and research ability among male nurses. Harmonious work passion stems from the autonomous internalization of work, making it part of the individual's self-identity and in harmony with other needs. Driven by intrinsic interest and values, employees with high harmonious passion are more likely to exhibit their true selves at work and pursue goals that align with their self-identity. Our data analysis showed that harmonious work passion positively correlates with research ability scores and its six dimensions. The high harmonious work passion group scored significantly higher in research ability than the low harmonious work passion group. This suggests that a passion for harmonious work may positively impact the research ability of male nurses. We speculate that this may be due to harmonious work passion enhancing male nurses' intrinsic motivation and sense of achievement in their work [24, 25, 28]. This positive emotional experience can inspire them to invest more energy and time into their work, thereby enhancing their creativity and research ability [23, 27].

Harmonious work passion reflects the consistency of intrinsic motivation, while obsessive work passion shows mixed motivational characteristics, where intrinsic motivation coexists with a sense of control from internal or interpersonal pressure [50]. Therefore, employees with high obsessive passion may be driven by both intrinsic motivation and a sense of responsibility or the desire to meet others' expectations. For obsessive work passion, we found that it also positively correlates with total research ability and its six dimensions. The scores of the low obsessive passion group were significantly lower than those of the high obsessive passion group. This indicates that obsessive work passion can also promote research ability to some extent, but its underlying mechanisms may differ from those of harmonious work passion. Obsessive work passion might drive individuals to invest more time and energy into work, despite this investment being accompanied by higher stress and anxiety. Male nurses with high obsessive passion may feel compelled to complete research tasks due to internal or external pressures, thus showing higher research ability in the short term [51–53]. However, this stress-driven enhancement of research ability may be unstable and can lead to job burnout and mental health issues [40, 41]. Furthermore, obsessive work passion may cause male nurses to focus more on the outcome of task completion rather than the learning and growth during the process. This outcome-oriented attitude may limit innovative thinking and the development of long-term research literacy, which is detrimental to the long-term development of scientific research. Moreover, obsessive work passion might affect male nurses' teamwork and job satisfaction. Due to high stress and anxiety, individuals with high obsessive passion may find it harder to establish good cooperative relationships with colleagues, potentially impacting the overall efficiency and effectiveness of research work. In contrast, harmonious work passion is more conducive to fostering teamwork and innovative thinking. Therefore, although obsessive work passion might improve research ability in the short term, in the long run, nursing managers should focus more on fostering harmonious work passion to ensure male nurses maintain efficiency in scientific research while sustaining good mental health and career development [54, 55].

To identify the main obstacles male nurses face in scientific research, we conducted a questionnaire survey. The statistics showed that male nurses consider the selection of research topics, design, writing, and funding constraints as the primary barriers to conducting nursing research. This might be due to the immature development of the nursing research field in China, where undergraduate nursing education focuses more on professional skills rather than research ability. Male nurses generally lack relevant experience and guidance, making it difficult to find innovative and practical research topics. Many male nurses lack systematic research training and find it hard to formulate scientific research plans. Additionally, many male nurses struggle to obtain sufficient financial support during their research, limiting the scale and depth of research and affecting access to research equipment and resources. To address these obstacles, we recommend strengthening the training of research abilities in nursing education and training, integrating research-related courses into the undergraduate curriculum, and providing relevant training for employed nurses. By offering systematic courses on research methods and practical opportunities, male nurses can improve their abilities in topic selection, design, and writing. Nursing managers should also actively seek research funding to provide more financial support to male nurses, alleviating their economic pressure during research. Moreover, establishing a mentorship system or research teams can provide more academic guidance and support, helping male nurses better conduct research. Only by overcoming these barriers can male nurses' research abilities be effectively enhanced, promoting the continuous development of the nursing discipline.

In summary, this study is the first to systematically explore the relationship between work passion and research ability among male nurses, revealing the different impacts of harmonious passion and obsessive passion on research ability. Our findings suggest that although obsessive work passion can improve research ability in the short term, fostering harmonious work passion is more beneficial for male nurses' research ability and mental health in the long run. We hope this study can provide valuable references for nursing managers and policymakers, helping them better support and motivate male nurses in their work, thus advancing nursing research and meeting the increasingly diverse nursing needs in clinical practice.

### 4.1. Limitation

While this study provides insights into the association between work passion and research ability among male nurses, there are some limitations should be noted.

Geographical constraints: The data were collected exclusively from Heibei province in China. Although we included 87 hospitals, regional variations in healthcare systems and nursing education may limit the generalizability of findings to other provinces or countries.

Sampling methodology: Although our samples come from 87 hospitals in Hebei Province, the coverage range is relatively wide in Hebei Province. The nonrandom convenience sampling approach, while pragmatic for multicenter data collection, may introduce selection bias. Future studies should employ stratified random sampling and increase sample size as much as possible to enhance representativeness. Besides, the variable selection do not include other variables that maybe potentially influence work passion, such as the number of night shifts and economic income, which could impact the analysis results. Therefore, future research can further explore more factors that may affect work enthusiasm. And it would be valuable to further discuss potential gender differences in this relationship.

## Figures and Tables

**Table 1:** Summary of participants' demographic information and scales.

Characteristics	Mean (SD)	Min–Max	Cutoff scores
Age (years)	1.95 (1.05)	1–5	—
Educational background	2.78 (0.47)	1–5	—
Entire period of actual operation	3.01 (1.35)	1–5	—
Job title	2.38 (0.79)	1–5	—
Academic part-time job status	1.50 (1.06)	1–5	—
Research project hosting status	1.24 (0.69)	1–5	—
Published papers status	1.37 (0.87)	1–5	—
Academic journal reviewer status	1.09 (0.47)	1–5	—
Academic exchange participation status	1.55 (0.99)	1–5	—
Technological awards status	1.15 (0.59)	1–5	—
Problem finding ability	7.74 (2.56)	0–12	—
Literature review ability	11.73 (4.66)	0–20	—
Research and design ability	9.31 (5.61)	0–20	—
Scientific research practice ability	12.15 (6.58)	0–24	—
Data processing ability	9.62 (5.55)	0–20	—
Paper writing ability	11.29 (6.95)	0–24	—
NRCS	61.85 (28.92)	0–120	—
Harmonious passion	38.27 (9.59)	7–49	> 39 (high), ≤ 39 (low)
Compulsive passion	27.75 (8.80)	7–49	> 28 (high), ≤ 28 (low)
WPS	66.02 (14.98)	20–98	≥ 67 (high), < 67 (low)

Abbreviations: NRCS, Nurse Research Capacity Scale; WPS, Working Passion Scale.

**Table 2:** Summary of the analysis of correlations among the scales (*r*).

	(1)	(2)	(3)	(4)	(5)	(6)	(7)	(8)	(9)	(10)
Problem finding ability (1)	1									
Literature review ability (2)	0.752^∗∗∗^	1								
Research and design ability (3)	0.624^∗∗∗^	0.803^∗∗∗^	1							
Scientific research practice ability (4)	0.684^∗∗∗^	0.784^∗∗∗^	0.879^∗∗∗^	1						
Data processing ability (5)	0.575^∗∗∗^	0.729^∗∗∗^	0.833^∗∗∗^	0.863^∗∗∗^	1					
Paper writing ability (6)	0.590^∗∗∗^	0.707^∗∗∗^	0.786^∗∗∗^	0.838^∗∗∗^	0.858^∗∗∗^	1				
NRCS (7)	0.739^∗∗∗^	0.872^∗∗∗^	0.928^∗∗∗^	0.952^∗∗∗^	0.925^∗∗∗^	0.915^∗∗∗^	1			
Harmonious passion (8)	0.481^∗∗∗^	0.463^∗∗∗^	0.359^∗∗∗^	0.380^∗∗∗^	0.362^∗∗∗^	0.382^∗∗∗^	0.434^∗∗∗^	1		
Compulsive passion (9)	0.217^∗∗∗^	0.165^∗∗^	0.140^∗∗^	0.194^∗∗∗^	0.154^∗∗^	0.150^∗∗^	0.183^∗∗∗^	0.326^∗∗∗^	1	
WPS (10)	0.435^∗∗∗^	0.393^∗∗∗^	0.312^∗∗∗^	0.357^∗∗∗^	0.322^∗∗∗^	0.333^∗∗∗^	0.386^∗∗∗^	0.832^∗∗∗^	0.796^∗∗∗^	1

*Note:* Calculated using 2-tailed bivariate correlations.

Abbreviations: NRCS, Nurse Research Capacity Scale; WPS, Working Passion Scale.

^∗∗^ *p* < 0.01.

^∗∗∗^ *p* < 0.001.

**Table 3:** Summary of total NRCS scores by one-way ANOVA test.

Characteristics	*N* (%)	Mean (SD)	*F*	*p*
Academic part-time job status			1.242	0.060
None	349 (79.68)	90.27 (29.14)		
Other committee member	12 (2.74)	100.93 (24.77)		
Municipal committee member and above	31 (7.08)	87.24 (22.65)		
Provincial committee members and above	39 (8.90)	101.62 (28.73)		
National committee members and above	7 (1.60)	121.28 (26.44)		
Research project hosting status			1.654	< 0.001^∗∗∗^
None	383 (87.44)	90.03 (28.94)		
Internal unit level	18 (4.11)	93.67 (24.43)		
Municipal level	25 (5.71)	109.97 (21.81)		
Provincial level	11 (2.51)	106.99 (30.94)		
National level	1 (0.23)	140.00 (0)		
Published papers status			1.066	0.321
None	355 (81.05)	90.83 (29.30)		
Other journal	33 (7.53)	94.08 (26.90)		
Science and technology core journal of China	28 (6.39)	90.81 (25.72)		
Chinese core journal of China	15 (3.42)	96.96 (24.67)		
SCI journal	7 (1.60)	126.29 (21.39)		
Academic journal reviewer status			1.395	0.008^∗∗^
None	418 (95.43)	91.35 (29.08)		
Other journal	9 (2.05)	106.76 (25.34)		
Science and technology core journal of China	4 (0.91)	85.53 (10.61)		
Chinese core journal of China	5 (1.14)	102.24 (23.64)		
SCI journal	2 (0.46)	116.50 (33.23)		
Academic exchange participation status			1.042	0.380
None	325 (74.20)	90.32 (30.00)		
Other	21 (4.79)	95.57 (17.24)		
Provincial	60 (13.70)	92.19 (25.89)		
National	30 (6.85)	103.93 (26.60)		
International	2 (0.46)	111.00 (41.01)		
Technological awards status			1.958	< 0.001^∗∗∗^
None	405 (92.47)	90.88 (28.80)		
Internal unit	13 (2.97)	93.01 (28.85)		
Municipal	10 (2.28)	109.30 (19.44)		
Provincial	7 (1.60)	119.41 (35.41)		

*Note:* Calculated using 2-tailed bivariate correlations.

Abbreviation: NRCS, Nurse Research Capacity Scale.

^∗∗^ *p* < 0.01.

^∗∗∗^ *p* < 0.001.

**Table 4:** Summary of total NRCS scores across multiple groups of demographic information by nonparametric tests.

Characteristics	*N* (%)	*H*	*p*
Age (years)		7.403	0.116
Under 30	194 (44.29)		
31–35	121 (27.63)		
36–40	79 (18.04)		
41–50	38 (8.68)		
Over 50	6 (1.37)		
Educational background		10.060	0.039^∗^
Below associate degree	3 (0.68)		
Associate degree	96 (21.92)		
Bachelor degree	334 (76.26)		
Master degree	3 (0.68)		
Doctor degree	2 (0.46)		
Entire period of actual operation (years)		11.003	0.027^∗^
Less than 3	89 (20.32)		
3–5	67 (15.30)		
5–10	98 (22.37)		
10–15	120 (27.40)		
More than 15	64 (14.61)		
Job title		4.607	0.330
None	45 (10.27)		
Junior title	218 (49.77)		
Medium-grade title	144 (32.88)		
Associate senior title	25 (5.71)		
Senior title	5 (1.14)		
Hospital level		8.333	0.040^∗^
Tertiary hospitals	280 (63.93)		
Secondary hospitals	150 (34.25)		
Hospitals below secondary	8 (1.83)		
Other health-related units	8 (1.83)		

*Note:* Calculated using 2-tailed bivariate correlations.

Abbreviation: NRCS, Nurse Research Capacity Scale.

^∗^ *p* < 0.05.

## Data Availability

The data that support the findings of this study are available from the corresponding author upon reasonable request.

## General Statement

We support inclusive, diverse, and equitable conduct of research.
